# Diuretic Treatment in Patients with Heart Failure: Current Evidence and Future Directions – Part I: Loop Diuretics

**DOI:** 10.1007/s11897-024-00643-3

**Published:** 2024-01-19

**Authors:** Joseph James Cuthbert, Andrew L Clark

**Affiliations:** 1 Clinical Sciences Centre, Hull York Medical School, University of Hull, Cottingham Road, Kingston-Upon-Hull, East Yorkshire, UK; 2 Department of Cardiology, Castle Hill Hospital, Hull University Teaching Hospitals Trust, Castle Road, Cottingham, East Yorkshire UK

**Keywords:** Diuretic treatment, Combination therapy, Loop diuretic, Decompensated HF

## Abstract

**Purpose of Review:**

Fluid retention or congestion is a major cause of symptoms, poor quality of life, and adverse outcome in patients with heart failure (HF). Despite advances in disease-modifying therapy, the mainstay of treatment for congestion—loop diuretics—has remained largely unchanged for 50 years. In these two articles (part I: loop diuretics and part II: combination therapy), we will review the history of diuretic treatment and the current trial evidence for different diuretic strategies and explore potential future directions of research.

**Recent Findings:**

We will assess recent trials including DOSE, TRANSFORM, ADVOR, CLOROTIC, OSPREY-AHF, and PUSH-AHF amongst others, and assess how these may influence current practice and future research.

**Summary:**

There are few data on which to base diuretic therapy in clinical practice. The most robust evidence is for high dose loop diuretic treatment over low-dose treatment for patients admitted to hospital with HF, yet this is not reflected in guidelines. There is an urgent need for more and better research on different diuretic strategies in patients with HF.

## Introduction

Fluid retention leading to peripheral and pulmonary congestion is the hallmark of symptomatic heart failure (HF) and is associated with poor quality of life and adverse outcome regardless of left ventricular ejection fraction (LVEF) [1]. Most patients admitted to hospital with HF have severe venous congestion (anasarca); the treatment for which is diuretics [2–4].

The majority of out-patients with chronic HF (and all patients admitted to hospital with HF) are treated with diuretics [5], but there is little consensus on the specific goals of diuretic therapy [6, 7]. The European Society of Cardiology HF guidelines recommend that “persistent congestion” is excluded before discharge in patients admitted to hospital with HF [6, 8]. However, many patients are discharged with residual congestion[9], including in some clinical trials [10•], and signs of congestion on ultrasound are common in the absence of signs on examination [11, 12].

But what is the point of achieving decongestion? The National Institute of Health and Care Excellence (NICE) guidelines state that diuretics should be used to improve symptoms [13], whereas the American Heart Association (AHA) guidelines recommend diuretics to improve symptoms *and* prognosis [14]. Aside from one meta-analysis of small placebo-controlled trials before the modern era of neuro-hormonal inhibition in HF [15], there are scant data to support either position.

There has only been a handful of modern-day trials of diuretics and diuretic strategies. Few have been powered to detect differences in outcome, and none has shown convincing symptomatic benefit. The “standard care” arm in each of the studies has been highly variable, often using low doses of intravenous (IV) furosemide. Perhaps as a result, the gold standard of treatment for fluid retention in HF (loop diuretic)—the dose of which often determined by physician preference—has remained unchanged for the last 60 years [16]. Here, we review the evidence to date for current practice of treatment with loop diuretics, and, in a separate article, examine future trends and possibilities.

## A Short History of Diuretic Treatment

The first detailed account of diuretic therapy for patients with HF was in 1785 [17]. William Withering reported improvements in breathing and peripheral oedema in patients with ‘dropsy’ (anasarca) who were treated with the leaves of *Digitalis purpurea*. Withering noted increased urine output as a side effect of treatment rather than a mechanism by which the patients were improving [6•]. Approximately 20 years later, the diuretic effects of mercury salts when given to patients with syphilis were described [18].

Digoxin and mercurial diuretics remained the only effective treatments for HF [19], until the discovery of acetazolamide [20], spironolactone [21], and thiazide diuretics in the 1950s [22]. All three were less toxic and better tolerated than mercury salts which quickly fell out of fashion [23, 24]. A decade later, 4-chloro-N-(2-furyl-methyl)-5-sulphmoyl anthranillic acid, or furosemide, was discovered, marking the last major advance in diuretic therapy for patients with HF [16].

Early trials showed that oral or IV administration of furosemide (and other loop diuretics) in patients with HF caused diuresis and was associated with improved symptoms compared to placebo [25–28]. Loop diuretics led to a larger and quicker diuresis than either acetazolamide, or spironolactone alone, and had an additive diuretic effect when given in combination with either agent (Table 1) [16, 29].

**Table 1:** Early trial studies of diuretic treatment/agents in patients with heart failure

Trial	Treatments	*N*	Population	Design and timeframe	Findings
Friedberg et al. [20]	Acetazolamide vs. no treatment	11	In-patients	Open-label, crossover	Greater diuresis and natriuresis with acetazolamide vs. control
Hanley et al. (1956)	Acetazolamide vs. mersalyl†	15	In-patients	Open-label, crossover, RCT; 3 days	Greater diuresis and natriuresis with acetazolamide vs. mersalyl and control
Schreiner et al. [22]	CTZ	20	In-patients	Case series	Increased diuresis, natriuresis, and weight loss in all patients
Carruthers et al. [21]	Spironolactone plus CTZ / HCTZ vs. CTZ or HCTZ alone	6	In-patients	Case series	Modest diuretic effect with the addition of spironolactone in some but not all patients
Stokes [16]	Furosemide, BFZ, chlorthalidone, and Aldactone	4	In-patients	Case series	Greater diuresis with furosemide vs. BFZ, chlorthalidone, or Aldactone. Greater diuresis with furosemide plus HCTZ, Aldactone, or chlorthalidone vs. either agent alone
Stewart et al. [31]	Furosemide vs. ethacrynic acid vs. BFZ vs. mersalyl† vs. spironolactone vs. no treatment	4	In-patients	Open-label, crossover, RCT; 24 days	Greater diuresis and natriuresis with furosemide vs. any other agent. Greater diuresis, natriuresis, and weight loss with all agents vs. no treatment
Stason et al. [29]	Furosemide, CTZ, ACZ, and meralluride†	46	7 healthy controls; 39 with oedema	Unblinded, non-randomised	Greater diuresis, natriuresis, and weight loss with furosemide vs. any other diuretic agent. Greater diuresis and natriuresis with furosemide plus another diuretic agent vs. furosemide alone
Kourouklis et al. [25]; Part II	Bumetanide vs. furosemide vs. placebo	10	In-patients	Cross-over 14 days	Similar weight loss, urine volume, and urinary electrolyte loss with an equivalent loop diuretic. Greater weight loss and urine volume with diuretic compared to placebo
Levy [32]	Furosemide vs. HCTZ plus spironolactone	21	Out-patients	Double-blind, RCT. 16 weeks	Greater diuresis with furosemide but no difference in symptoms or serum electrolytes
Coodley et al. [33]	Furosemide vs. diapAmide vs. no treatment	30	In-patients	Open-label, cross-over, RCT	Similar natriuresis but greater diuresis with furosemide vs. diapAmide. Greater diuresis with both active treatments compared to no treatment
Gabriel et al. [34]	Furosemide vs. bumetanide vs. BFZ	18	Out-patients	Open-label, cross-over, RCT	No difference in weight loss but greater potassium loss with BFZ vs. either loop diuretic
Sherman et al. [26]	Piretanide vs. placebo	38	In-patients	Double-blind, RCT; 28 days	Greater diuresis, weight loss, and improvements in HF symptoms and signs with diuretic vs. placebo
Crawford et al. [30]	Furosemide plus amilioride vs. cyclopenthiazide plus potassium chloride	47	Out-patients with HF	Open-label, RCT	Greater improvement in symptoms with furosemide-amilioride combination than with thiazide-potassium chloride combination (*P* < 0.05)
Haerer et al. [27]	Piretanide vs. placebo	60	Out-patients	Single-blind, non-randomised; 21 days	Weight loss, improved symptoms, increased exercise tolerance with diuretic. No change with placebo
Kleber et al. [28]	Ibopamine vs. HCTZ vs. ibopamine + HCTZ vs. placebo	247	Out-patients	Double-bind, RCT; 8 weeks	Ibopamine + HCTZ caused greater weight loss than HCTZ alone. All active treatments caused greater weight loss than placebo. Greater rate of hypokalaemia in patients receiving HCTZ either alone or in combination

Abbreviations: *BFZ* bendroflumethiazide, *HCTZ* hydrochlorothiazide, *CTZ* chlorothiazide, *ACZ* acetazolamide

^†^mercurial diuretic

Compared to thiazide diuretics, loop diuretics tended to induce a greater diuresis and greater symptomatic improvement, with a lower risk of hypotension, hypokalaemia, or hyponatraemia [30–35]. Amiloride and triamterene inhibit sodium–potassium co-transporters in the distal convoluted tubule, which increases sodium excretion and reduces potassium excretion—‘potassium-sparing’ diuretics [36, 37]. Both were used only in combination with loop or thiazide diuretics to counter the excess potassium loss seen in early studies [38]. They have been superseded by spironolactone which has a similar (mild) potassium-sparing diuretic effect but profound prognostic benefits in patients with HF and a reduced ejection fraction (HeFREF) [39].

Loop diuretics have remained the cornerstone of the treatment of venous congestion in patients with HF since the 1960s. However, there is very little evidence to support their use [15], and even less on which to base recommendations on dosing or administration.

## Loop Diuretics

**Fig. 1:**
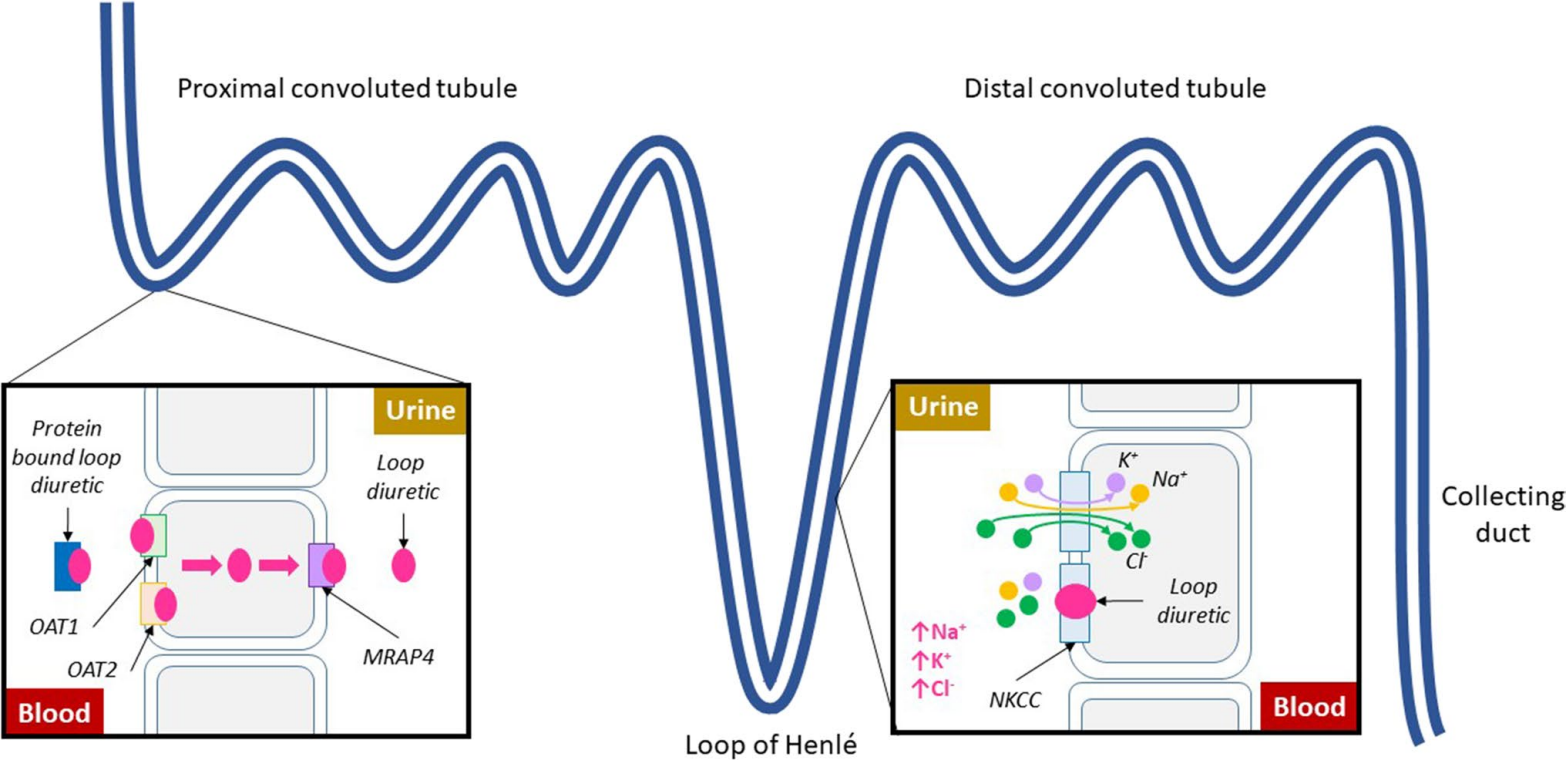
Mechanism of action of loop diuretics. Abbreviations OAT, organic anion transporter; MRAP, multidrug resistance-associated protein

Loop diuretics are organic anions and are highly protein-bound in the serum [16]. They are actively excreted into the urinary space, entering the basolateral membrane of cells in the proximal convoluted tubule (PCT) through organic anion transporters 1 and 2 (OAT1 and OAT2) and are moved into the urinary space via the multidrug resistance-associated protein 4 (MRAP4) (Fig. 1) [40]. They compete with chloride ions to bind to and inhibit the sodium–potassium-chloride co-transporter (NKCC) on the apical membrane of the thick ascending limb of the loop of Henle. The NKCC reabsorbs filtered Na^+^, K^+^, and Cl^−^ ions in a 1:1:2 ratio [41]. Inhibition of the NKCC thus increases the urinary concentrations of each electrolyte and reduces the ion concentration in the renal medulla, causing natriuresis and diuresis [42].

Common side effects or complications of loop diuretics include renal dysfunction [43] and electrolyte abnormalities (hyponatraemia, hypokalaemia, hypochloraemia, and metabolic alkalosis) [44–47], all of which are associated with worse outcomes. In particular, renal dysfunction and hypochloraemia (amongst other mechanisms) may contribute to loop diuretic resistance [48, 49]. Renin release is governed, in part, by urinary sodium and chloride concentrations detected in the PCT by NKCC co-transporters in the macula densa (MD). By inhibiting the NKCC2 in the MD, loop diuretics increase activation of the renin–angiotensin–aldosterone system (RAAS) [50–52]. While activation of the RAAS is commonly thought to result from abnormal haemodynamics in patients with HF, plasma concentrations of renin and aldosterone may be normal in patients with symptomatic HF who are not taking diuretics, increasing only after loop diuretics have been started: [53, 54] it is possible that loop diuretics are the primary driver of renin-angiotensin aldosterone system (RAAS) activation in patients with HF.

### Which Loop Diuretic to Use?

Furosemide is the most commonly used loop diuretic in both in- and out-patients [55, 56]. However, there are important and, possibly, clinically significant differences in the pharmacokinetics of oral furosemide, bumetanide, and torsemide (Table 2) [29, 57, 58].

**Table 2:** Pharmacokinetic properties of oral loop diuretics

	Furosemide	Bumetanide	Torasemide
Half-life (minutes)	90–120	60	180–240
*Renal dysfunction*	170	100	240–300
*Heart failure*	160	80	360
Onset (minutes)	30–60	30–60	30–60
Bioavailability (%)	10–100	80–100	80–100

There have been a few head-to-head comparisons of loop diuretics in patients with HF. Small, open-label RCTs with short-term follow-up found few differences in symptoms or diuresis between furosemide and bumetanide [59–61]. However, others suggested that mortality, hospitalisation, symptoms, and quality of life were better with torsemide compared to furosemide [62–64]; a definitive trial followed (Table 3).

**Table 3:** Randomised head-to-head trials comparing different kinds of loop diuretic agents

Trial	Treatments	*N*	Population	Design and timeframe	Findings
Konecke [61]	Furosemide vs. bumetanide	42	Out-patients	Open-label, RCT; 16 weeks	No difference in symptoms, diuresis, blood pressure, renal function, or serum electrolytes
Sagar et al. [60]	Furosemide vs. bumetanide	30	Out-patients	Double-blind, RCT; 7 days	No difference in diuresis or serum electrolytes
Ramsey et al. [58]	Furosemide-Amiloride vs. bumetanide-potassium chloride	40	Out-patients	Open-label, RCT; 8 weeks	Trend towards improvement in symptoms and peripheral oedema with bumetanide but no statistical difference
Murray et al. [64]	Furosemide vs. torasemide	234	Out-patients	Open-label, RCT; 12 months	Lower rate of HF hospitalisation with torasemide vs. furosemide (17% vs. 32%; *P* < 0.01). Greater improvement in fatigue with torasemide but no difference in other symptoms
TORIC [63]	Furosemide vs. torasemide	1377	Out-patients	Open-label, non-randomised. 12 months	Improved symptoms and lower mortality (2.2 vs. 4.5%; *P* < 0.05) with torasemide vs. furosemide
Muller et al. [62]	Furosemide vs. torasemide	237	Out-patients	Open-label, RCT; 9 months	Greater symptomatic and quality of life improvement with torasemide vs. furosemide
TRANSFORM (2023)	Furosemide vs. torasemide	2859	Out-patients; 65 years; 70% HeFREF; NTproBNP 3994 ng/L	Open-label, RCT; median 17-month follow-up	No difference in all-cause hospitalisation (RR 0.94 (95% CI 0.84–1.07), or all-cause mortality (26.1% vs. 26.2%; HR = 1.02 (95% CI 0.89–1.18)

Abbreviations: *RCT* randomised controlled trial, *HF* heart failure, *HeFREF* HF with a reduced ejection fraction, *NTproBNP* N-terminal pro-B-type natriuretic peptide, *RR* rate ratio, *HR* hazard ratio, *CI* confidence interval

The TRANSFORM (Effect of Torsemide vs Furosemide After Discharge on All-Cause Mortality in Patients Hospitalized With Heart Failure) trial randomised 2859 patients to either oral furosemide or torsemide at the point of discharge following admission with HF, the dose of which was determined by the treating clinician [65•]. Most patients had HeFREF (70%) and were taking a loop diuretic prior to admission (66%). The primary endpoint was all-cause mortality, and the secondary endpoints included all-cause mortality or all-cause hospitalisation. Most patients were taking 80 mg of oral furosemide equivalents per day (the mean dose of oral loop diuretic on discharge was 79 mg of furosemide equivalents in both arms).

There was no difference in either the primary or secondary endpoint during a median follow-up of 17 months. Perhaps, the most notable finding of TRANSFORM was the dire prognosis following a hospitalisation with HF: 26% of patients in each arm died after a median follow-up of 17 months, and nearly half of patients had either died or were re-admitted in the first year following discharge.

There was no benefit of one treatment over the other in sub-group analysis. No data was collected on symptoms, diuresis, or congestion at different time points, so we do not know if the prognosis was poor due to inadequate diuresis, or residual congestion at the point of discharge. Regardless, given the high event rate and neutral findings, it is probable that the type of loop diuretic used is not important [66].

### What Dose to Use?

Not all patients with HF need diuretic treatment. Optimal medical therapy may negate the need for loop diuretic treatment in some patients with HeFREF. Angiotensin-converting enzyme inhibitors [67], sacubitril valsartan [68], mineralocorticoid receptor antagonists [69], and sodium-glucose co-transporter 2 inhibitors [70], all have either a mild diuretic effect or reduce the need for loop diuretic treatment.

Diuretic withdrawal in patients with no or minimal congestion who are receiving optimal disease-modifying therapy is associated with improvements in renal function and reductions in plasma renin concentration without worsening symptoms or an increased risk of hospitalisation during short-term follow-up [71, 72]. However, diuretic withdrawal will not be suitable for all euvolaemic patients.

One small study of medication withdrawal (including diuretics) in stable out-patients with HF and a reduced ejection fraction (HeFREF) found worsening symptoms and doubling of serum natriuretic peptide concentration after 48 h [73]. In the only randomised controlled trial of diuretic withdrawal in stable out-patients with HeFREF, all of whom had NYHA class I symptoms and were receiving less than 80 mg of furosemide equivalents per day, 1 in 4 needed to restart loop diuretic during 90-day follow-up [74]. For those without congestion, the optimal dose may be that which prevents the recurrence of congestion, which will vary between patients and may change over time.

Dosing of furosemide for patients with congestion, either oral or IV, is contentious. Observational studies have found that higher doses are associated with the worse outcome both in patients hospitalised with HF [75, 76] and out-patients with chronic HF [1, 55, 77]. However, these analyses are confounded by the need to treat more severe disease with higher doses of diuretic.

Some observational data suggest that loop diuretics are associated with improved survival in those with more severe congestion [78, 79] and that increasing the dose of loop diuretic is associated with improved survival, regardless of the dose used, *if* the severity of congestion also improves [80].

For patients hospitalised with HF who were taking a loop diuretic prior to admission, the ESC and AHA guidelines recommend starting patients on 1–2 × their usual daily dose of oral diuretics in divided doses given IV [8, 14]. The NICE guidelines for acute HF merely recommend IV doses “higher” than that of the oral dose on admission [81].

Most patients who are prescribed a loop diuretic take no more than 80 mg per day of oral furosemide equivalents [82–85], but up to 50% of patients are not taking a loop diuretic on admission [86, 87]. In these circumstances, guidelines recommend low doses of IV furosemide (20–40 mg per day) [8, 14]. Thus, the starting dose of IV furosemide for patients admitted to the hospital could range anywhere between 20 and 160 mg per day.

The Diuretic Optimisation Strategies Evaluation (DOSE) trial attempted to clarify the optimal diuretic dosing strategy for patients admitted to hospital with HF and the best mode of administration (bolus vs. continuous infusion) [88]. Patients were randomised to either low dose (usual dose of oral loop diuretic) vs. high dose (2.5 × usual dose of oral loop diuretic) *and* to either continuous infusion or twice daily bolus administration of loop diuretic (Table 4). The co-primary endpoints were the change in symptoms measured on a visual analogue scale of 0 to 100 and the change in serum creatinine from randomisation to 72 h.

**Table 4:** Trials of different strategies of loop diuretic administration

Trial (date)	Population	*N*	Strategy and treatments	Design and timeframe	Findings
DOSE (2011) Dosing	In-patients; 66 years; LVEF 35%; NTproBNP 7439 ng/L	308	High dose; − 258 mg per day vs. low dose; − 119 mg per day	Double-blind; multi-centre; 2 × 2 design; 1:1:1:1 randomisation to high dose (2.5 × pre-admission dose) or low dose (1 × pre-admission dose) and BD bolus dosing or continuous infusion; 72 h	Greater weight loss (3.9 kg vs. 2.8 kg; *P* = 0.01), greater net fluid loss (4.9 L vs. 3.6 L; *P* = 0.001), and greater improvement in breathlessness (*P* = 0.04) at 72 h with high vs. low dose. Greater chance of changing to oral diuretics and less chance of increasing the dose of IV diuretics at 48 h with high vs. low dose. No difference in length of hospitalisation or days alive out-of-hospital at 60 days. Higher adverse event rate in low-dose arm
DOSE (2011) Strategy			BD bolus dosing vs. continuous infusion		No difference in symptoms, diuresis, weight loss, or adverse events. Greater chance of requiring an increase in loop diuretic dose (21% vs. 11%; *P* = 0.01) or needing the addition of a thiazide diuretic (25% vs. 11%; *P* = 0.02) after 48 h with bolus dosing vs. continuous infusion
PUSH-AHF (2023)	In-patients 74 years LVEF 35% NTproBNP 4390 ng/L	310	Natriuresis guided dosing of diuretic; − 160 mg per day vs. standard care; − 80 mg per day	Open-label; single centre; 1:1 randomisation to natriuretic-guided treatment or standard care	Greater natriuresis at 24 h (409 mmol vs. 345 mmol; *P* = 0.006) and 48 h (653 mmol vs. 575 mmol; *P* = 0.02). Greater diuresis at 24 h (3900 mL vs. 3300 mL; *P* = 0.005) and 48 h (6655 mL vs. 5915 mL; *P* = 0.01). No difference in either natriuresis or diuresis at 72 h. Patients in the natriuretic-guided arm received twice as much diuretic during hospitalisation than did patients in the standard care arm

Abbreviations: *LVEF* left ventricular ejection fraction, *NTproBNP* N-terminal pro-B-type natriuretic peptide, *BD* twice daily, *IV* intravenous

The trial was neutral in that neither dosing nor administration strategy was superior in respect to the primary endpoints, but there were a number of interesting, clinically relevant findings (Table 4):

1. Patients in the high-dose arm (median daily dose 258 mg) had greater weight loss, greater net fluid loss, and greater improvement in breathlessness than patients in the low-dose arm (median daily dose 119 mg) after 72 h.
2. Patients in the high-dose arm were more likely to change to oral diuretics and less likely to require intensification of diuretic treatment at 48 h than were patients in the low-dose arm
3. Despite the greater diuresis with high-dose diuretic compared to low dose, the adverse event rate was *higher* in the low-dose arm.
4. Patients in the bolus dosing arm were approximately twice as likely to require either an increase in loop diuretic dose or the addition of a thiazide diuretic at 48 h as patients in the continuous infusion arm. Despite these differences, there was no difference in weight loss, fluid loss, freedom from congestion, or improvement in symptoms between continuous or bolus dosing.

The results suggest that (1) high-dose treatment (2.5 × the oral dose on admission) induces a greater diuresis than does low-dose treatment, without a greater risk of adverse effects and (2) that continuous infusion may cause a similar diuresis to bolus dosing but without the need for treatment intensification. Due to the short half-life of IV loop diuretics, bolus dosing may allow for a period between doses during which renal sodium resorption may increase (and diuresis decrease) [89]. Other studies have found a greater diuresis and greater improvements in clinical congestion with continuous infusions over bolus dosing [90, 91].

Concerns over side effects or complications of high-dose IV loop diuretic are commonplace but may lead to an over-cautious approach to diuretic treatment. In the CARESS trial of ultrafiltration (UF) versus diuretic therapy for patients admitted to hospital with HF and worsening renal function, the diuretic therapy arm allowed for up to 720 mg of IV furosemide per day plus 10 mg of metolazone per day. The co-primary endpoint was a change in serum creatinine and a change in weight at 96 h. Both groups lost a similar amount of weight, but renal function improved in the diuretic therapy arm, and hyponatraemia or hypokalaemia occurred in only 3% of patients. Although not powered to detect a difference in clinical outcome, there was a trend towards a higher rate of all-cause hospitalisation or death in the UF arm [92].

Surprisingly, the DOSE trial has had little influence on guidelines or clinical trial protocols which continue to allow bolus administration of low doses (doses equal to the oral dose taken on admission) and, in some cases, discourage the use of continuous infusions [93].

Perhaps, the most useful lessons of the DOSE and TRANSFORM studies are that it is not the type of loop diuretic you use that matters, but what you do with it (in terms of dosing and administration strategy) that counts.

## Strategies of Loop Diuretic Dosing and Administration

**Fig. 2:**
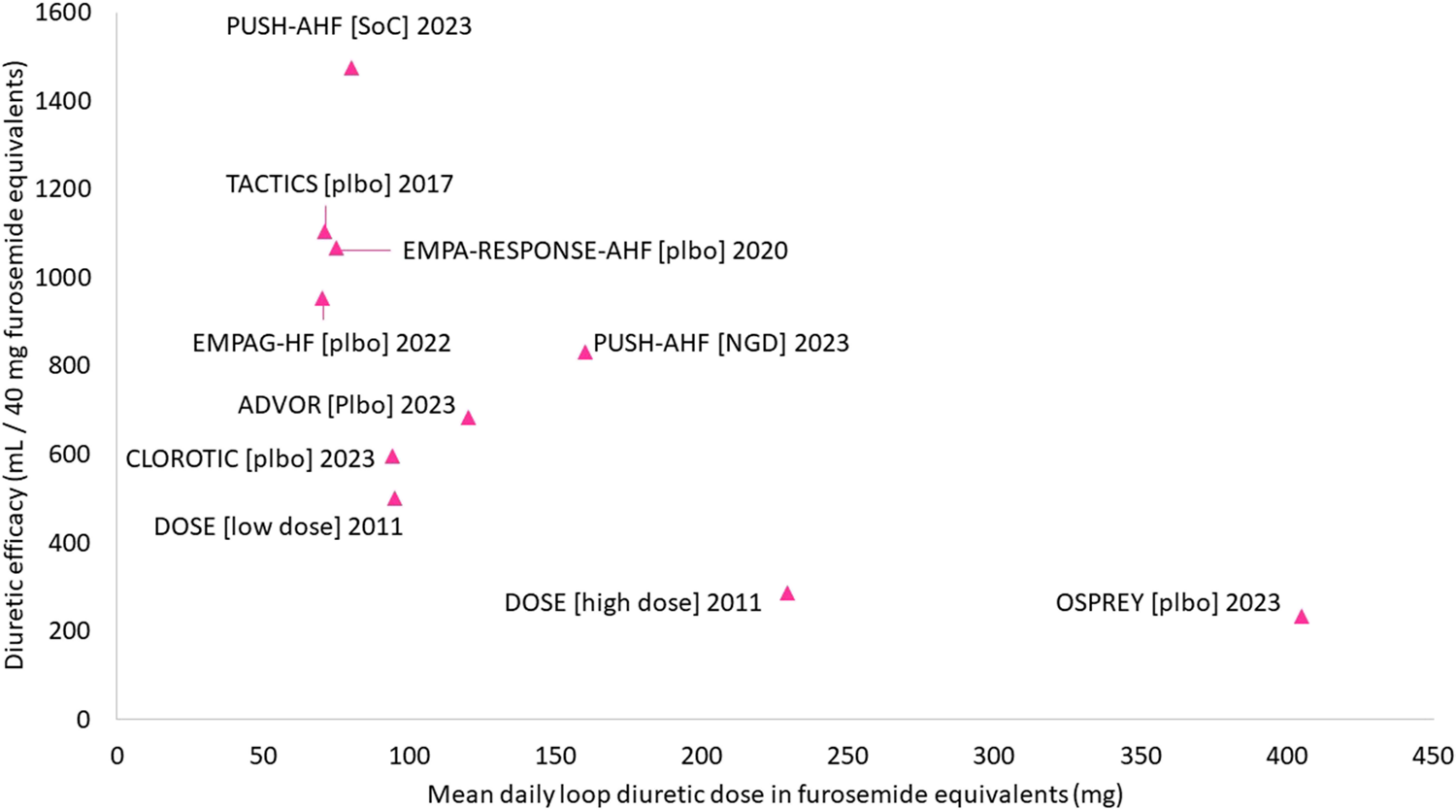
Loop diuretic efficacy in recent diuretic trials. Daily furosemide equivalents reported in text in DOSE, ADVOR, and TACTICS trials and estimated from reported cumulative dose in CLOROTIC, EMPA-RESPONSE-AHF, EMPAG-HF, PUSH-AHF, and OSPREY. Daily urine output reported in PUSH-AHF, CLOROTIC, and EMPA-RESPONSE-AHF trials; estimated from reported cumulative urine output at 72 h in the DOSE and TACTICS trials and at 96 h in the OSPREY trial; estimated from figures in the ADVOR and EMPAG trials

Some have suggested that patients respond to loop diuretics in an ‘all-or-none’ fashion, with conditions such as chronic kidney disease causing some patients to have a higher threshold to induce diuresis than others [94]. While all trial data to date demonstrate this is as a gross over-simplification, the data indicate that response to treatment is subject to a law of diminishing returns: doses above a certain threshold do not induce a much greater diuresis, and loop diuretic efficacy (volume of urine produced per 40 mg of furosemide) [95] falls with increasing doses (Fig. 2) [96]. Identifying patients unlikely to respond to diuretic therapy early during treatment may allow early titration of treatment in some and avoid the use of excessively high doses in others.

### Natriuresis-Guided Loop Diuretic Treatment

Poor ‘natriuretic response’ (low urine sodium concentration after administration of IV loop diuretic) is associated with a greater risk of worsening renal function, inadequate treatment of congestion, loop diuretic resistance, and poor prognosis in patients treated with IV loop diuretic [97–99]. Natriuretic-guided dosing of loop diuretics—increasing dose of loop diuretic to achieve a given urine sodium concentration—has featured in ESC HF recommendations since 2019, but the efficacy of natriuresis-guided diuretic treatment was not assessed in an RCT until 2023 [8].

In the PUSH-AHF trial, 310 patients admitted to the hospital with HF were randomised to either natriuresis-guided therapy or standard care [100•]. In both arms, the initial dose of loop diuretic was based on renal function and prior loop diuretic use and was given as a bolus. Patients randomised to the natriuresis-guided arm had urine sodium concentration tested at 2, 6, 12, 18, and 24 h. The bolus dose was doubled (with a maximum dose of 5 mg bumetanide (200 mg furosemide)) at any time point if there was poor natriuretic response (defined as urinary sodium concentration < 70 mmol/L). The primary endpoint was total natriuresis after 24 h of treatment (Table 4).

Unsurprisingly, titrating loop diuretic dose based on urine sodium concentration was associated with greater natriuresis compared to standard therapy. Urine output at 24 and 48 h was also greater in the natriuresis-guided arm. This, again, was unsurprising. Eighty-five percent of patients in the natriuresis-guided arm had their diuretic dose doubled during the first 24 h, and, despite similar median initial dose of a loop diuretic (160 mg furosemide equivalents in both arms), patients in the natriuresis-guided arm received almost twice as much diuretic during admission as those in the standard care arm (26 mg of bumetanide over 7-day hospitalisation period, ~ 4 mg per day = 160 mg furosemide equivalents vs. 15 mg bumetanide over 7-day hospitalisation, and ~ 2 mg per day = 80 mg furosemide per day).

Natriuresis-guided treatment was stopped after 24 h, and, perhaps as a result, the differences in natriuresis and urine output were lost after 72 h of treatment. There was no difference in the adverse event rate, length of hospitalisation, re-admission with HF, or mortality between the two groups. Although hypotension is a concern when giving bolus doses of up to 200 mg IV furosemide, these data were not reported. The non-randomised ENACT-HF trial reported similar results which have been presented but not published [101].

**Fig. 3:**
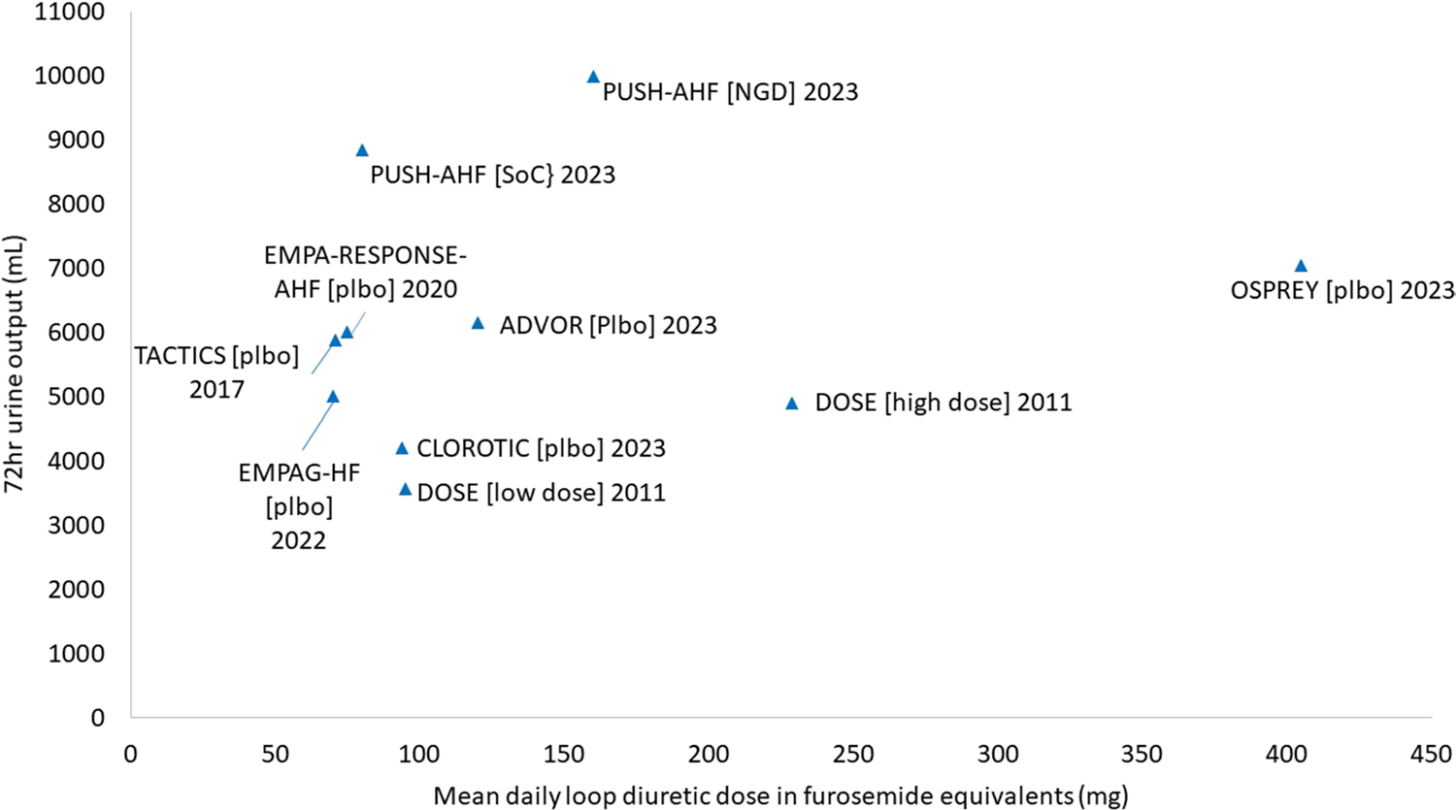
Seventy-two-hour urine output in diuretic trials. Daily furosemide equivalents reported in text in the DOSE, ADVOR, and TACTICS trials and estimated from reported cumulative dose in the CLOROTIC, EMPA-RESPONSE-AHF, EMPAG-HF, PUSH-AHF, and OSPREY trials. Seventy-two-hour urine output reported in the DOSE and TACTICS trials; estimated from urine output reported at 24 h in the PUSH-AHF, CLOROTIC, and EMPA-RESPONSE-AHF trials, at 48 h in the ADVOR trial, and at 96 h in the OSPREY trial; estimated from figures in the EMPAG trial

The practicalities of repeated testing of urine sodium concentration in a busy ward environment aside, the PUSH-AHF and ENACT-HF trials demonstrate (as the DOSE trial did a decade earlier) that the larger the dose of loop diuretic you give, the greater the diuresis you induce (Fig. 3). Only a minority of patients had an adequate natriuretic response to the initial dose of diuretic, even though approximately half were not taking loop diuretic prior to admission. These trials, and natriuretic-guided diuretic treatment as a strategy, do not advance our understanding of how to use diuretics. It is unlikely that natriuresis-guided treatment will become the standard care for most HF specialists in busy healthcare systems.

### Out-patient Parenteral Loop Diuretic Treatment

Administering IV loop diuretic to ambulatory out-patients is a common practice in the USA, and there is an increasing use of so-called ‘furosemide lounges’ (whereby patients attend an ambulatory unit, are cannulated, given an IV bolus of loop diuretic, and return home) in Europe. Avoiding hospitalisation has obvious benefits for the patient and healthcare systems, but supporting data are almost non-existent. Some observational reports suggest that either IV or subcutaneous (SC) furosemide given either at home or in a furosemide lounge, can cause weight loss, improve symptoms and signs of congestion, and reduce admission to hospital in most, but not all patients [102–105].

There has been one small RCT of treatment in a furosemide lounge versus conventional in-patient care (*N* = 24) in patients deemed to require at least 2 days of IV loop diuretic therapy. After 60 days, patients treated in the furosemide lounge accrued numerically more days alive and out-of-hospital compared to those treated as an in-patient (47 vs. 59; *P* = 0.13). However, more patients treated in the furosemide lounge were readmitted after 60 days (6 vs. 2; *P* = 0.31) [106]. No firm conclusions can be drawn from the small sample size, and a larger trial is planned [107].

SC furosemide infusions are commonly used in patients with advanced HF and palliative care needs for whom hospitalisation is unsuitable or unwanted [108]. Data from small randomised studies suggest that subcutaneous furosemide may have a similar diuretic effect to IV furosemide [109–111]: larger studies are planned (EudraCT 2020–004833-19) [112].

However, caution is required when interpreting trials of out-patient strategies. Days alive out-of-hospital at a given point in time may be an appealing primary endpoint [102, 112], but it is problematic. Randomising patients to out-patient parenteral treatment immediately shortens the length of the index hospitalisation. The median length of stay in a hospital in the UK is 8 days [4], and patients randomised to out-patient treatment will get a head start. If patients require longer treatment in the out-patient setting than they do as an in-patient due to insufficient diuresis, the days spent out-of-hospital will be greater, but disability due to symptoms, and the inconvenience of increased urine output, health care visits, and treatment equipment will be prolonged. Another problem is that any potential harm of early discharge may take longer than 30 days to manifest; days alive and out-of-hospital may be similar or greater in the early discharge arm at more distant time points.

Treatment in the out-patient setting is only safe if patients are able to cope with the increased burdens of transport, increased urine output at home, and the equipment for IV or SC furosemide infusions. Patients who are deemed unable to cope are likely to be excluded from the trials. The vast majority of patients admitted to hospital with HF are aged over 75, with moderate to severe peripheral oedema, NYHA class III or IV symptoms, and multiple co-morbidities [4]. It is unclear what proportion of patients admitted to hospital with HF would be suitable for parenteral treatment in the out-patient setting. Eligibility may depend far more on social circumstances than on patient or disease characteristics.

Finally, there are very few data comparing parenteral diuretic therapy to increased oral therapy. In the only head-to-head RCT in patients with HF (*N* = 10), there was a little difference in the urine output 8 h after treatment between those receiving 80 mg furosemide subcutaneously or orally (1550 mL (range 1353 to 1866 ml) vs. 1833 ml (range 1623 to 2726 ml), respectively, *P* not reported) [110]. Whether increasing oral diuretic is as effective as parenteral treatment given at home is unknown but may be preferable from a health economic and patient convenience point-of-view.

## Summary and Conclusion

In 1964, the English physician Wilfred Stokes who used up to 300 mg of furosemide per day wrote that ‘Dosage [of furosemide] is largely arbitrary, governed by limits of known safety, experience, and recommendation.’ [16] Over half a century later, very little has changed.

The results of the DOSE trial are largely ignored by clinical guidelines but demonstrate that high-dose loop diuretic given via a continuous infusion induces a greater diuresis without the need for treatment intensification than does low-dose treatment. Natriuretic-guided dosing of diuretic in PUSH-AHF also found that most patients fail to respond to a low-dose diuretic (even if loop diuretic naïve) and that giving higher doses induces a greater diuresis without an increase in adverse events.

Hospitalisation with severe fluid retention is a common cause of morbidity in patients with HF and for some, high-dose loop diuretic treatment alone may be sufficient. However, hospitalisation often lasts many days, at great cost to the patient and healthcare system. Early discharge and use of parenteral treatment at home or in a furosemide lounge may shorten hospital stay but may only be suitable for a minority of patients. Data from randomised trials on the safety and efficacy of the furosemide lounge is non-existent. An alternative approach may be to speed up in-patient diuresis using combination therapy—loop diuretic plus an adjunctive diuretic treatment—which we will consider in part 2 of this review.

## Data Availability

No datasets were generated or analysed during the current study.
